# Revascularization of human ovarian cortical grafts is not equally efficient from both sides of the cortex tissue

**DOI:** 10.3389/fendo.2025.1679248

**Published:** 2025-11-28

**Authors:** Jiaojiao Cheng, Xiangyan Ruan, Juan Du, Fengyu Jin, Muqing Gu, Jing Jin, Yanglu Li, Yanqiu Li, Zecheng Wang, Lingling Jiang, Mingzhen Zhang, Anming Liu, Alfred O. Mueck

**Affiliations:** 1 Department of Gynecological Endocrinology, Beijing Obstetrics and Gynecology Hospital, Capital Medical University, Beijing Maternal and Child Health Care Hospital, Beijing, China; 2 Department for Women's Health, University Women's Hospital and Research Centre for Women's Health, University Hospitals of Tuebingen, Tuebingen, Germany

**Keywords:** ovarian tissue cryopreservation and transplantation, revascularization, proliferation, fertility preservation, ovarian cortical grafts, hormone restoration

## Abstract

**Research question:**

Does early-phase revascularization of human ovarian cortical grafts exhibit spatial asymmetry between medullary and cortical surfaces in a xenotransplantation model?

**Design:**

Cryopreserved ovarian tissue from five patients was transplanted bilaterally beneath the renal capsule of immunodeficient nude mice (medullary surface oriented downward). 10 ovariectomized mice (OVX) without transplantation served as controls. Grafts and blood were collected on post-transplantation days 3 and 7 (n=5 mice/point). Blood samples were also collected from OVX mice at the same time points (n=5 mice/point) for evaluation of estradiol (E2) and follicle-stimulating hormone (FSH). Vascular density (CD31^+^/CD34^+^), cellular proliferation (Ki67^+^), and apoptosis (TUNEL^+^) were quantified via immunohistochemistry at graft interfaces.

**Results:**

CD31^+^ density was significantly higher at the medullary interface compared to the cortical interface at day 3 (217.25 ± 17.65 *vs.* 79.15 ± 14.10; *P* < 0.001) and day 7 (279.63 ± 22.65 *vs.* 197.32 ± 16.08; *P* = 0.002). CD34^+^ density showed similar medullary predominance at day 3 (149.32 ± 12.98 *vs.* 72.01 ± 15.48; *P* = 0.001) and day 7 (300.57 ± 24.65 *vs.* 238.35 ± 22.44; *P* = 0.010). Medullary vascularity increased significantly from day 3 to 7 (CD31^+^: *P* = 0.014; CD34^+^: *P* < 0.001). Cortical vascular density demonstrated significant time-dependent augmentation (*P* < 0.001 for both markers). Ki67^+^ cell percentages showed no significant differences between surfaces at day 3 (medullary *vs.* cortical 55.78% ± 5.05% *vs.* 56.48% ± 4.61%; *P* = 0.924) or day 7 (53.76% ± 4.65% *vs.* 61.80% ± 5.35%; *P* = 0.246). Apoptosis was significantly lower at the medullary interface than cortical interface at day 3 (4.19% ± 0.86% *vs.* 18.83% ± 4.05%, *P* = 0.001) and day 7 (0.40% ± 0.09% *vs.* 3.98% ± 1.10%, *P* = 0.000), and decreased markedly over time at both sites. Ovarian tissue transplantation significantly elevated E2 levels and suppressed FSH levels compared to the OVX controls.

**Conclusions:**

Human ovarian grafts demonstrated superior revascularization and significantly reduced apoptosis at the medullary interface compared to the cortical surface. The restoration of hormone levels confirmed functional graft survival, validating the experimental model. These findings underscore the significance of surgical orientation in facilitating graft revascularization and mitigating cellular stress during early ovarian tissue transplantation.

## 1 Introduction

Ovarian tissue cryopreservation (OTC) followed by transplantation (OTCT) represents an established fertility preservation strategy for female cancer patients, as endorsed by guidelines (1, 2). This approach constitutes the sole option for prepubertal girls and women requiring immediate gonadotoxic therapy (3–6). Over 200 live births have been reported worldwide following OTCT until 2021 (7, 8), with the majority resulting from spontaneous conception after orthotopic transplantation (3). Restoration of ovarian endocrine function and follicular development occurs in 70%-95% of recipients, achieving pregnancy and live birth rates of 44% and 19%-32%, respectively (2).

While patient age at OTC influences outcomes, determinants of success remain incompletely characterized (7). The transplantation site represents a critical factor. A multicenter analysis of 285 OTCT procedures detailed placement onto decorticated ovarian medulla (16.7%), newly created peritoneal windows (62.7%), or both sites (20.4%) (7). Orthotopic transplantation demonstrates superior efficacy in restoring endocrine and reproductive function compared to other sites (7, 9).

However, pelvic damage from prior surgery or radiotherapy may preclude orthotopic transplantation, necessitating heterotopic sites. Reported experience with heterotopic transplantation remains limited (10). Xenograft study suggests that non-kidney heterotopic sites (e.g., murine ear/muscle) exhibit increased fibrosis and reduced follicle numbers relative to the kidney capsule grafts (10). To date, only one live birth has been achieved following transplantation to an abdominal sub-peritoneal pocket, resulting in twin delivery after *in vitro* fertilization and embryo transplantation (11). Additionally, Gook et al. documented a live birth originating from a metaphase I oocyte retrieved from an 8mm follicle, which underwent *in vitro* maturation and successful fertilization (10). Suboptimal temperature at subcutaneous sites and mechanical constraints may impair follicular development, though adequate conditions can support full cortical function (10).

A critical limitation of OTCT is early graft hypoxia and ischemia-reperfusion injury due to the absence of vascular anastomosis, which can precipitate the loss of up to 90% of follicles and curtail graft longevity (12, 13). While revascularization of human ovarian grafts demonstrates equivalent efficiency across cortical surfaces by 8 weeks post-transplantation (14), spatial dynamics of revascularization during the critical early phase (≤7 days) remain undefined. Using immunodeficient mice helps overcome some limitations of traditional transplantation methods by avoiding immune rejection, thereby allowing the development and survival of transplanted tissue. Revascularization is critical for the survival and function of grafted ovarian tissue (15). This study therefore evaluates the spatiotemporal patterns of angiogenesis (assessed via CD31/CD34), cellular proliferation (Ki67), and apoptosis (Terminal deoxynucleotidyl transferase dUTP Nick-End Labeling, TUNEL) across the cortical and medullary surfaces of human ovarian tissue grafts during the early post-transplantation period (3 and 7 days) in a mouse kidney capsule model. The recovery of endocrine function was also assessed through plasma estradiol (E2) and follicle-stimulating hormone (FSH) measurements. This study aims to provide insights into graft survival and potential clinical applications.

## 2 Materials and methods

### 2.1 Experimental design

Cryopreserved human ovarian cortical tissue from five patients (20-31 years) was thawed and xenotransplanted bilaterally beneath the renal capsules of immunodeficient nude mice (n=10 total; Sibeifu Biotechnology Co., Beijing, SCXK[Jing]2019-0010). An additional group of 10 mice underwent bilateral ovariectomy (OVX) without subsequent transplantation to serve as controls. All animals were housed under controlled conditions (20-22 °C, 12h light/dark cycle) with free access to food and water. All experimental procedures were approved by the Committee on Animal Research of the Capital Medical University (AEEI-2020-064; May 6, 2020).

**Figure 1:**
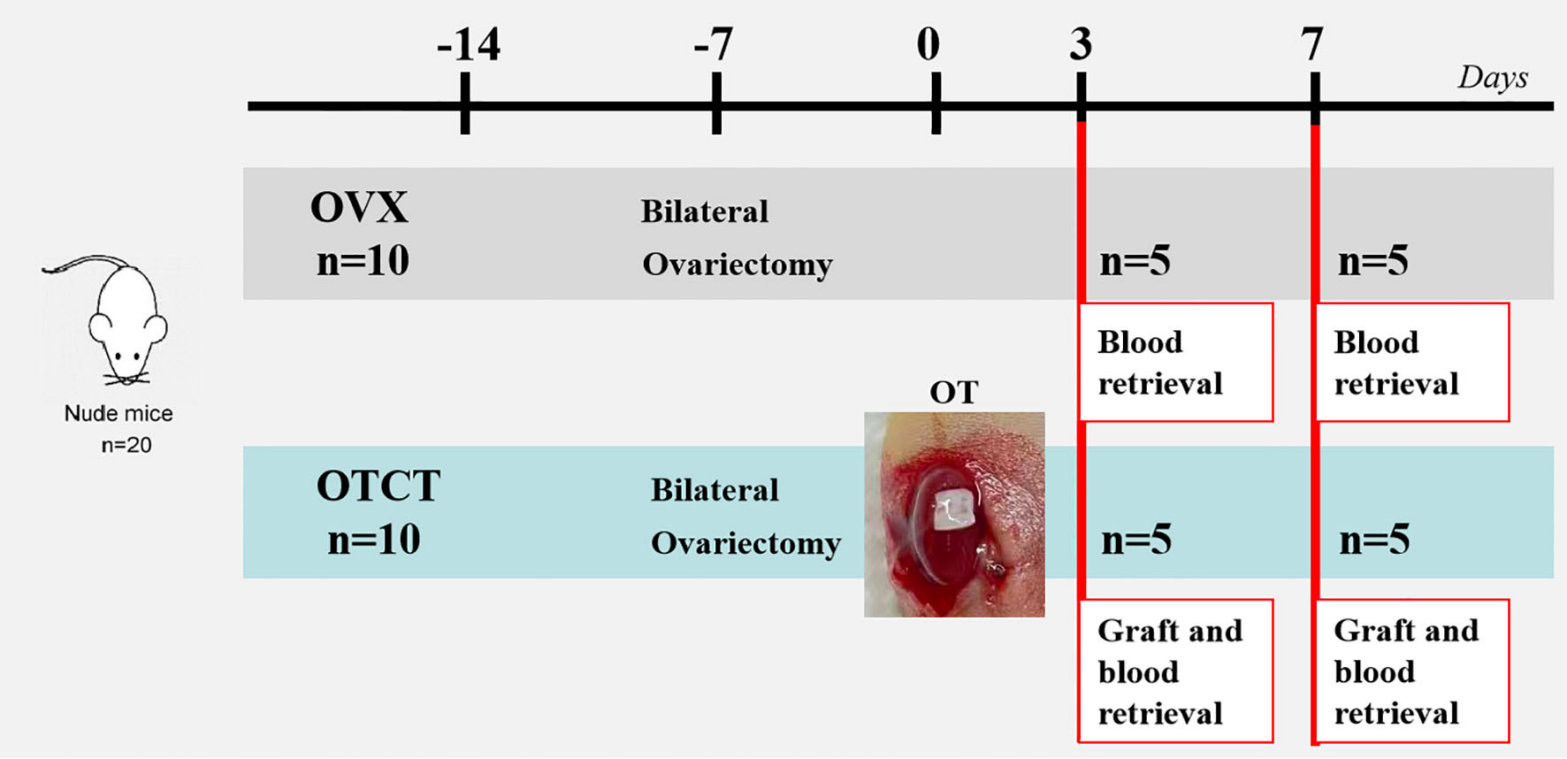
Experimental design. Ovarian tissue from 5 patients was transplanted to the renal capsule of 10 mice. Another 10 mice underwent bilateral ovariectomy without subsequent transplantation to serve as controls. Mice were euthanized on days 3 and 7, and samples were collected for histological and hormone analyses. OVX, bilateral ovariectomy; OT, ovarian transplantation; OTCT, ovarian tissue cryopreservation and transplantation.

After one week of acclimatization, bilateral ovariectomy was performed under sodium pentobarbital anesthesia (40 mg/kg, i.p.). One weeks post-ovariectomy, thawed ovarian cortical fragments were transplanted bilaterally beneath the renal capsule of immunodeficient nude mice (n=10, medullary surface oriented downward). Grafts and blood samples were collected on post-transplantation days 3 (n = 5 mice, 10 grafts) and 7 (n = 5 mice, 10 grafts), a timeframe selected to capture the critical phase of graft revascularization, as referenced in previous work (16). Blood samples were also collected from OVX mice at the same time points (n = 5 mice per time point) for subsequent E2 and FSH analysis (**Figure 1**).

### 2.2 Ovarian tissue cryopreservation and thawing procedure

Human tissue use was approved by the Beijing Obstetrics and Gynecology Hospital, Capital Medical University Ethics Committee (2020-KY-007-01; April 20, 2020). Twenty cortical fragments (3mm×3mm×1mm) from five breast cancer patients were obtained from our cryobank following written research authorization. In accordance with standardized OTC protocols, a uniform thickness of 1 mm was maintained for all cortical strips, reflecting the fact that the human follicular reserve is predominantly located within the outer 1 mm of the cortex (17–19). Follicle density per 3mm fragment was quantified using Calcein-AM (146, 50, 45, 95, 84, respectively) (20). Tissue processing involved: OTC: slow-freezing protocol (6); Thawing: Sequential sucrose dilution (21): Room temperature (1 min) →37°C water bath (2 min); Stepwise transfer through decreasing sucrose concentrations.

### 2.3 Transplantation technique

All surgical procedures were performed under strict aseptic conditions. A left subcostal incision was made to expose the kidney. Using a 25G needle, the renal capsule was carefully micro-dissected to create a subcapsular pocket. The thawed human ovarian cortical graft, oriented with its medullary surface facing downward, was then inserted subcapsularly without the use of sutures or other fixation methods. Following graft placement, the kidney was gently repositioned into the abdominal cavity, and the muscle layer and skin incisions were closed using 5/0 absorbable polypropylene suture (Prolene^®^, Ethicon, Somerville, NJ, USA). An identical surgical procedure was subsequently performed on the right kidney to place the contralateral graft (22).

### 2.4 Immunohistochemical staining for graft vascularization and proliferation

Immunohistochemistry (IHC) was performed to assess graft vascularization and cellular proliferation using specific markers: CD31, CD34, and Ki67. For each harvested graft (*n* = 10 per time point), 20 consecutive sections were cut from both the cortical and medullary surfaces, with each section having a thickness of 5 μm. For each marker (CD31/CD34/Ki67), four sections from each surface were selected for staining, with an interval of 25 μm between adjacent sections for the same marker on the same surface.

CD31 is a well-established marker of mature endothelial cells and is widely used to identify functional blood vessels. The anti-CD31 antibody used in this study is a rabbit monoclonal anti-mouse CD31, thereby allowing specific detection of host-derived (mouse) vasculature within the graft. In contrast, CD34 is expressed on both mature endothelial cells and endothelial progenitor cells and is often associated with nascent and remodeling vasculature. The anti-CD34 antibody employed cross-reacts with both human and murine CD34, enabling the visualization of the total vascular network (including any pre-existing human vessels and newly formed mouse vessels) (23). This methodological approach was chosen to specifically address the early phase of graft revascularization, which is predominantly driven by the rapid ingrowth of host vessels to re-establish perfusion, as previously described (16).

The IHC protocol consisted of the following steps: antigen retrieval using Tris-EDTA buffer (pH 9.0, Servicebio G1203), blocking of endogenous peroxidase activity with 3% hydrogen peroxide (H_2_O_2_, Servicebio G0115), and blocking of non-specific protein binding with 3% bovine serum albumin (BSA, Servicebio G5001). Sections were then incubated overnight at 4 °C with primary antibodies: Ki67 (1:800 dilution, Servicebio GB111141), CD34 (1:500 dilution, Servicebio GB13013), and CD31 (1:500 dilution, Servicebio GB13428). Following primary incubation, sections were incubated for 60 minutes at room temperature with a horseradish peroxidase (HRP)-conjugated goat anti-rabbit secondary antibody (Servicebio G1211). Visualization was achieved using 3,3’-Diaminobenzidine (DAB) chromogen, followed by hematoxylin nuclear counterstaining. Appropriate controls were included: positive controls consisted of known human ovarian tissue sections, and negative controls were performed by omitting the primary antibody during incubation. Quantitative analysis was conducted on digitally scanned whole slides using a PANNORAMIC 250 scanner (3DHISTECH). Vascular density was quantified as the number of CD31^+^ or CD34^+^ structures per square millimeter (structures/mm²) by analyzing four non-overlapping fields per slide at 200× magnification using HALO image analysis software (v3.0.311.314, Indica Labs) (24). The proliferation index was calculated as the percentage of Ki67^+^ nuclei relative to the total number of nuclei counted. All quantitative assessments were performed independently by two investigators blinded to the experimental groups (25).

### 2.5 Apoptosis

TUNEL staining was carried out using the DeadEnd™ Fluorometric TUNEL System (Promega Corp., Madison, WI, USA) in accordance with the manufacturer’s protocol. Apoptosis was assessed on both cortical and medullary surfaces. For each time point and surface, four sections spaced at 25 μm intervals were selected for staining, following the methodology detailed in our previous work (24). All sections were automatically analyzed with QuantCenter 2.1 software to obtain counts of TUNEL (+) and DAPI-positive cells. The apoptotic rate of stromal cells was determined as the ratio of TUNEL (+) cells to DAPI-positive cells.

### 2.6 Hormonal assays

At the time of graft retrieval under anesthesia, blood samples were collected and allowed to clot at room temperature for 15 minutes. After centrifugation at 2,000 g for 20 min, the supernatant was collected and stored at -80°C. E2 and FSH were measured using specific commercial enzyme-linked immunosorbent assay kits (E2: ab108640; FSH: ab108678; both from Abcam, China), following the established protocol from our previous study (24). All measurements were performed in triplicate.

### 2.7 Statistical analysis

SPSS 23.0 (Chicago, IL, USA) was used for data analysis. Kolmogorov-Smirnov conducts the normality test of the data. Normally distributed data is represented by mean ± standard error of the mean (SEM). A comparison between groups is performed by one-way analysis of variance (ANOVA); non-normally distributed data are described by median and interquartile range. Two-way ANOVA with Tukey’s *post-hoc* test analyzed effects of time (3/7 days) and interface (medullary/cortical). Analyses employed SPSS 23.0 with graphing in GraphPad Prism 6.0. Significance thresholds: *P < 0.05, **P < 0.01, ***P < 0.001.

## 3 Results

### 3.1 Graft revascularization

**Figure 2:**
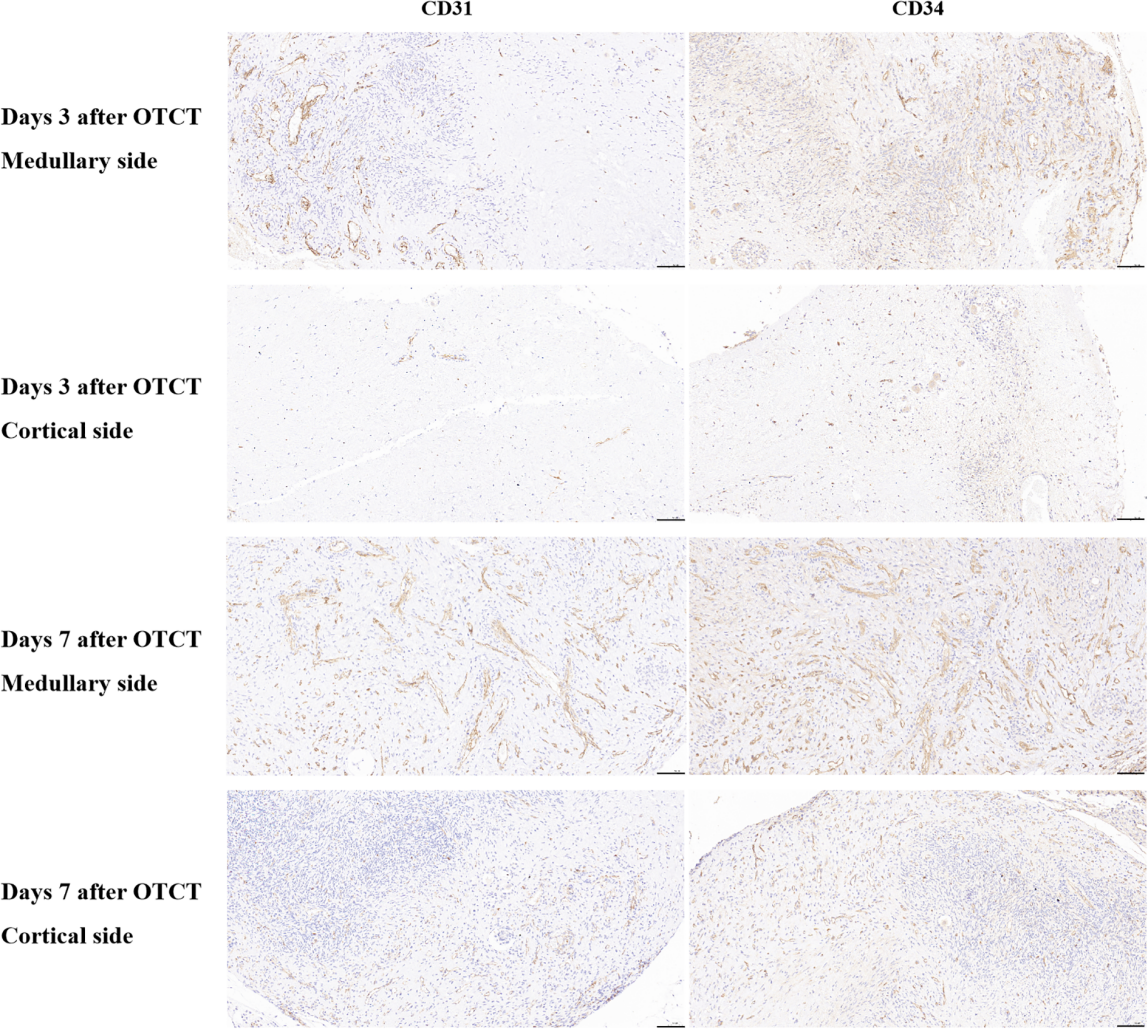
CD31, CD34 immunostaining in the medullary and cortical sides of human ovarian xenograft after 3 and 7 days of xenotransplantation. Scale bar: 100 μm. OTCT, ovarian tissue cryopreservation and transplantation.

**Figure 3:**
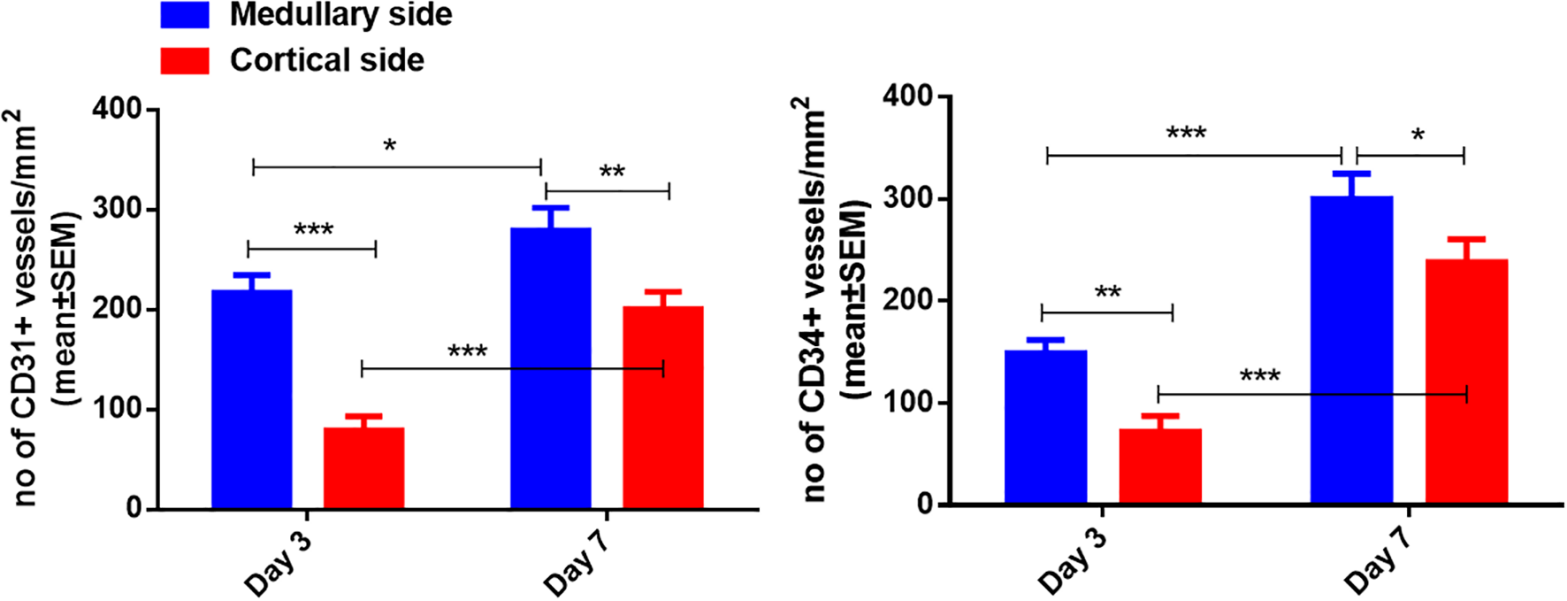
Quantification of CD31-positive and CD34-positive vessel density in the medullary and cortical sides of human ovarian xenograft. Significance thresholds: *P < 0.05, **P < 0.01, ***P < 0.001.

Human ovarian grafts exhibited significant spatial heterogeneity in revascularization during early transplantation. At both post-transplantation time points, vascular density (assessed by CD31^+^ and CD34^+^ structures) was markedly higher at the medullary interface compared to the cortical interface (**Figures 2**, **3**; **Table 1**): Day 3: CD31^+^ density: 217.25 ± 17.65 *vs.* 79.15 ± 14.10 (medullary *vs.* cortical; *P* < 0.001). CD34^+^ density: 149.32 ± 12.98 *vs.* 72.01 ± 15.48 (*P* = 0.001). Day 7: CD31^+^ density: 279.63 ± 22.65 *vs.* 197.32 ± 16.08 (*P* = 0.002). CD34^+^ density: 300.57 ± 24.65 *vs.* 238.35 ± 22.44 (*P* = 0.010).

**Table 1:** The no of CD31 (+), CD34 (+) vessels/mm^2^, percentage of Ki67 (+) cells, and percentage of TUNEL (+) cells in the medullary and cortical sides at 3 days and 7 days after OTCT.

Immunohistochemical staining index and TUNEL assay	Days 3 after OTCT (mean ± SEM)		Days 7 after OTCT (mean ± SEM)		*P* value (a *vs.* b)	*P* value (a *vs.* c)	*P* value(c *vs.* d)	*P* value(b *vs.* d)
	Medullary side (a)	Cortical side (b)	Medullary side (c)	Cortical side (d)				
no of CD31 (+) vessels/mm^2^	217.25 ± 17.65	79.15 ± 14.10	279.63 ± 22.65	197.32 ± 16.08	0.000	0.014	0.002	0.000
no of CD34 (+) vessels/mm^2^	149.32 ± 12.98	72.01 ± 15.48	300.57 ± 24.65	238.35 ± 22.44	0.001	0.000	0.010	0.000
percentage of Ki67 (+) cells (%)	55.78 ± 5.05	56.48 ± 4.61	53.76 ± 4.65	61.80 ± 5.35	0.924	0.785	0.246	0.440
Percentage of TUNEL (+) cells (%)	4.19 ± 0.86	18.83 ± 4.05	0.40 ± 0.09	3.98 ± 1.10	0.001	0.000	0.000	0.000

Furthermore, both vascular markers demonstrated significant temporal progression (*P* < 0.05 for all comparisons): Medullary interface showed increased CD31^+^ (+28.7%; *P* = 0.014) and CD34^+^ (+101.3%; *P* < 0.001) density from day 3 to 7. Cortical interface exhibited greater relative gains: CD31^+^ (+149.3%) and CD34^+^ (+231.0%) densities increased significantly over time (All *P* < 0.001).

### 3.2 Cellular proliferation

**Figure 4:**
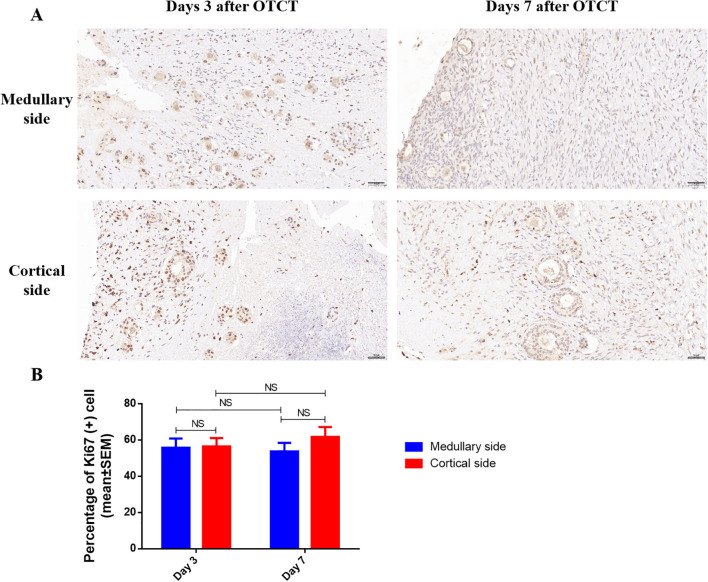
**(A)** Ki67 immunostaining in the medullary and cortical sides of human ovarian xenograft after 3 and 7 days of xenotransplantation. **(B)** Quantification of the percentage of Ki67-positive cells in the medullary and cortical sides of human ovarian xenograft. Scale bar: 50μm. NS, No significant; OTCT, ovarian tissue cryopreservation and transplantation.

Ki67^+^ proliferation indices remained comparable between medullary and cortical interfaces at both time points (**Figure 4**; **Table 1**): Day 3: 55.78% ± 5.05% *vs.* 56.48% ± 4.61% (*P* = 0.924). Day 7: 53.76% ± 4.65% *vs.* 61.80% ± 5.35% (*P* = 0.246). No significant temporal changes or spatial differences were detected (*P* = 0.861 for group-time interaction).

### 3.3 Cellular apoptosis

**Figure 5:**
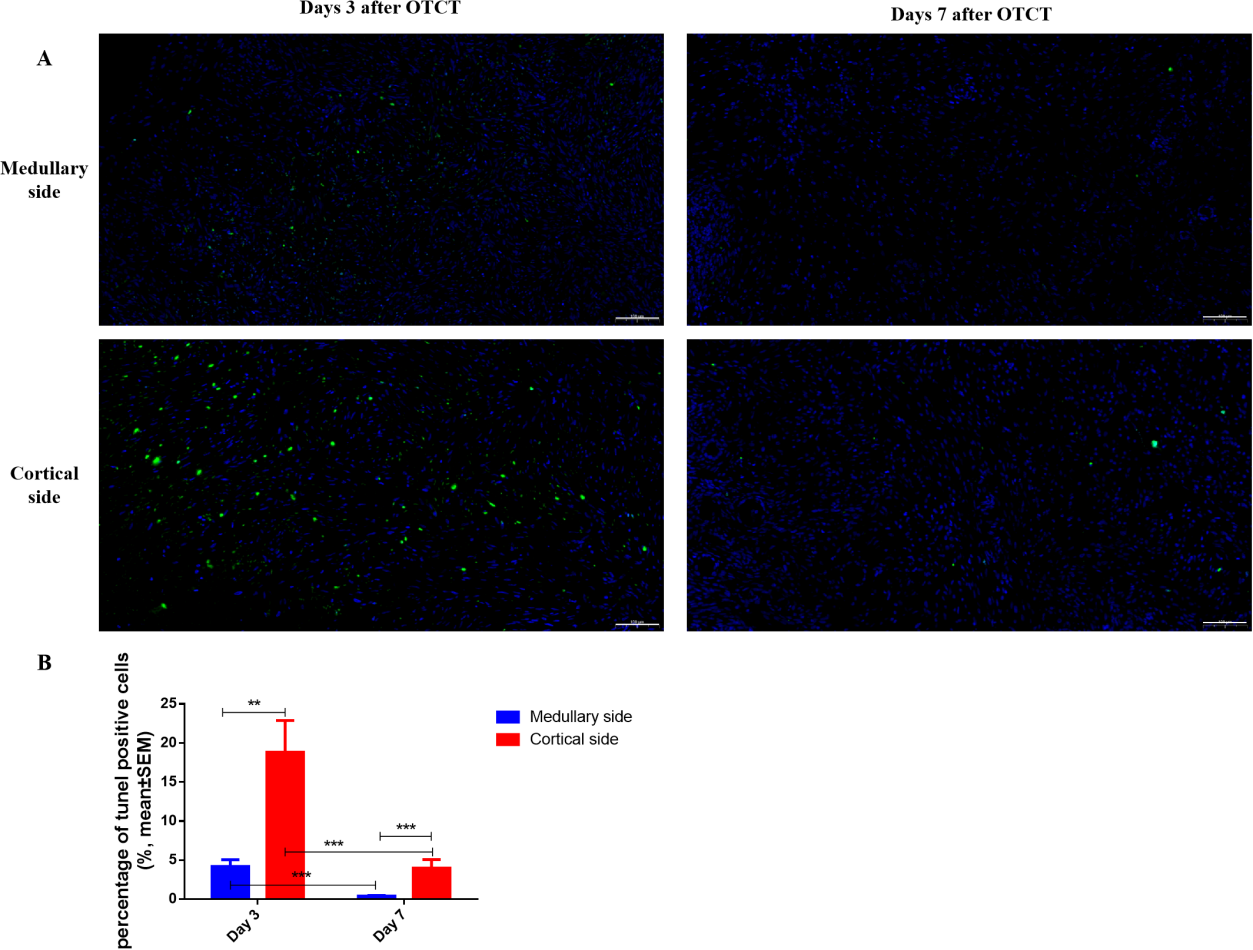
**(A)** TUNEL immunostaining in the medullary and cortical sides of human ovarian xenograft after 3 and 7 days of xenotransplantation. **(B)** Quantification of the percentage of TUNEL-positive cells in the medullary and cortical sides of human ovarian xenograft. Scale bar: 100μm. **P < 0.01, ***P < 0.001.

At both post-transplantation time points, the percentage of TUNEL (+) cells was significantly lower at the medullary interface compared to the cortical interface (**Figure 5**; **Table 1**): Day 3: the percentage of TUNEL (+) cells: (medullary *vs.* cortical; 4.19% ± 0.86% *vs.* 18.83% ± 4.05%, *P* = 0.001). Day 7: (0.40% ± 0.09% *vs.* 3.98% ± 1.10%, *P* = 0.000). Additionally, a significant reduction in apoptosis was observed from day 3 to day 7 at both the medullary and cortical interfaces (All *P* = 0.000).

### 3.4 E2 and FSH levels in mice’ plasma

**Figure 6:**
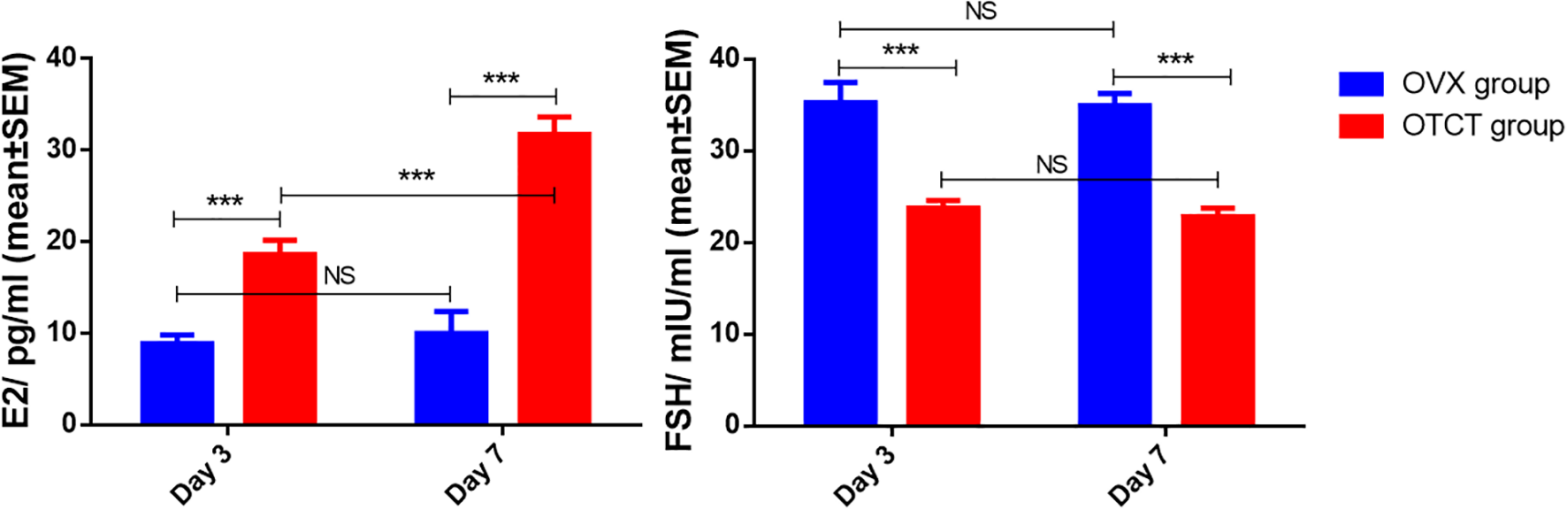
E2 and FSH concentration in mice’s plasma. ***P < 0.001. OVX, bilateral ovariectomy; OTCT, ovarian tissue cryopreservation and transplantation; E2, estradiol; FSH, follicle-stimulating hormone; NS, No significant.

Following OTCT, plasma E2 levels increased while FSH levels decreased compared to the OVX control group (**Figure 6**). Specifically, E2 levels in the OVX group were significantly lower than in the OTCT group on day 3 (8.87 ± 0.96 pg/ml *vs.* 18.60 ± 1.59 pg/ml, *P* = 0.000) and day 7 (9.97 ± 2.40 pg/ml *vs.* 31.74 ± 1.92 pg/ml, *P* = 0.000). Conversely, FSH concentrations in the OVX group were significantly higher than those in the OTCT group on both day 3 (35.29 ± 2.19 mIU/ml *vs.* 23.78 ± 0.85 mIU/ml, *P* = 0.000) and day 7 (34.95 ± 1.33 mIU/ml *vs.* 22.85 ± 0.93 mIU/ml, *P* = 0.000).

Within the OVX group, no significant differences were observed in E2 or FSH levels between day 7 and day 3 (*P* = 0.605 and *P* = 0.893, respectively). In the OTCT group, E2 levels were significantly higher on day 7 compared to day 3 (*P* = 0.000), whereas no significant difference was found in FSH levels between the two time points (*P* = 0.465).

## 4 Discussion

This study reveals a distinct site-specific pattern during the early revascularization phase of human ovarian cortical grafts: the medullary interface exhibited superior revascularization and significantly less apoptosis than the cortical surface shortly after transplantation. Although the cortical interface was initially less vascularized, it demonstrated considerable potential for vascular expansion over time. Revascularization proceeded independently of cellular proliferation, as indicated by comparable Ki67^+^ rates across both interfaces. Critically, the successful restoration of endocrine function - marked by elevated E2 and suppressed FSH levels in transplanted mice - confirmed the functional viability of the grafts. Together, these integrated findings regarding revascularization, apoptosis, proliferation, and hormonal recovery provide mechanistic insight into early graft survival and offer a scientific rationale for optimizing clinical transplantation strategies.

### 4.1 Early revascularization and reduced apoptosis of human ovarian cortex grafts

The presence of revascularization is crucial for the successful transplantation of ovarian grafts, as it facilitates the rapid establishment of the blood supply, which is essential for the survival of ovarian follicles (26, 27). The consistent superiority of medullary revascularization (CD31^+^/CD34^+^ density 2.7- to 2.1-fold higher than cortical at day 3). The 4.5-fold lower apoptotic rate at the medullary interface on day 3 (4.19% vs 18.83%), strongly suggests that rapid vascular establishment directly mitigates ischemic damage. This observation aligns with the structural gradient of human ovarian tissue, where the medulla’s inherent vascular richness and loose connective tissue architecture likely facilitate host vessel invasion. The presence of medulla in ovarian cortex is beneficial for post-transplantation development of cryopreserved human ovarian tissue (28). The progressive decrease in apoptosis at both interfaces from day 3 to day 7, coinciding with vascular maturation, further supports the crucial role of revascularization in stabilizing the graft. Our data extend Kristensen et al. (14) observation of equivalent late-phase revascularization by demonstrating that initial vessel ingrowth occurs preferentially through the medullary-capsular interface, thereby providing superior protection against apoptosis during the vulnerable early phase of transplantation (14). The significantly higher density of murine CD31-positive vessels at the medullary interface as early as day 3 underscores the superior capacity of this surface to attract and facilitate the ingrowth of host-derived vasculature, which is critical for initiating graft perfusion and reducing ischemic apoptosis during this vulnerable period.

When considering our findings within the broader paradigm of vascularization mechanisms—namely, angiogenesis (sprouting from pre-existing vessels) and vasculogenesis (*de novo* formation from progenitor cells)—the early and dominant presence of murine CD31-positive mature vessels strongly indicates that the observed revascularization is primarily driven by host-derived angiogenesis. This interpretation is supported by Van Eyck et al. (23), who demonstrated that the initial phase of graft revascularization is characterized by the ingrowth of host vessels, a classic angiogenic process. While CD34 can mark endothelial progenitor cells involved in vasculogenesis, its co-localization with the robust CD31 signal at these early time points suggests that the predominant process we observed is the angiogenesis of mature host vasculature. Therefore, the superior revascularization at the medullary interface can be more precisely defined as a heightened efficiency in initiating and facilitating host-derived angiogenesis.

The markedly superior revascularization observed at the medullary interface likely stems from its inherent structural and biological predisposition to angiogenesis. We hypothesize that the medulla’s richer native extracellular matrix (ECM) composition, particularly higher baseline levels of pro-angiogenic factors (e.g., VEGF, FGF) and a more permissive collagen architecture (e.g., higher type I/III ratio), facilitates more rapid and robust host vessel ingrowth and anastomosis compared to the denser, more compact cortical stroma. Furthermore, the medullary compartment naturally harbors a network of small vessels and stromal cells primed for vascular remodeling. Upon transplantation, this pre-existing “pro-angiogenic niche” may respond more efficiently to hypoxic stress, accelerating the recruitment and integration of host-derived endothelial cells. In contrast, the cortical surface, primarily designed for follicle nurturing within a tightly packed stromal environment, may lack this immediate vascular responsiveness, leading to the observed delay in neovascularization. Future studies specifically profiling the ECM and angiogenic factor gradients across these interfaces are warranted to validate this hypothesis.

### 4.2 Endocrine functional recovery of the graft

Notably, the cortical interface exhibited a 231.0% increase in CD34^+^ density between days 3-7 versus 101.3% at the medullary interface (*P* < 0.001). This compensatory angiogenesis suggests cortical tissue retains robust vascular plasticity, potentially mediated by hypoxia-induced VEGF upregulation (20). While medullary dominance persisted through day 7, the narrowing differential (CD31^+^ ratio: 2.7→1.4) indicates cortical revascularization is time-dependent rather than intrinsically deficient. This explains why prior studies examining later timepoints (e.g., Kristensen’s 8-week assessment) found no interface differences.

Functional recovery was further corroborated by temporal hormone dynamics. The steady increase in E2 levels from day 3 to day 7 within the OTCT group indicates progressively enhancing steroidogenic activity, consistent with ongoing graft maturation and vascular expansion. The absence of temporal hormonal changes in the OVX group confirms that these shifts are specifically driven by the grafted ovarian tissue.

Collectively, the restoration of ovarian endocrine function following transplantation provides the ultimate validation of graft success. The significantly elevated E2 levels and decreased FSH levels in transplanted mice compared to OVX controls demonstrate not only graft survival but also ovarian endocrine functional recovery. This hormonal profile confirms that the observed revascularization patterns translate to physiological functionality, reinforcing the clinical relevance of our findings. The correlation between medullary-oriented revascularization, reduced apoptosis, and restored ovarian endocrine function provides a comprehensive understanding of the graft recovery process.

### 4.3 Proliferation-vascularization decoupling

The absence of Ki67^+^ disparity between interfaces (*P* = 0.861) despite vascular gradients implies that: Early graft survival may rely more on diffusion than perfusion (16). Vascular endothelial cells rather than parenchymal cells drive initial proliferation (29). This decoupling highlights that optimizing angiogenesis – not just preserving follicular integrity – determines transplant success.

### 4.4 Clinical translation: bidirectional revascularization strategy

#### 4.4.1 Clinical strategy of OTCT

The transplantation method of the Donnez team (30) and the Silber team (31) is that the ovarian tissue graft is fixed to the remaining stripped ovary with sutures or Interceed ^®^, and the medulla is attached to the remaining ovary face down, to simulate the normal ovarian structure. The transplantation method of the Andersen team and the Meirow team is: longitudinal or transverse incision on the remaining intact ovary in the body (32–34), and ovarian tissue is transplanted under the ovarian cortex to promote revascularization on both sides (30). The Donnez/Silber medulla-down approach directly leverages our observed medullary revascularization superiority, while Andersen/Meirow’s subcortical placement capitalizes on cortical plasticity for bidirectional revascularization.

#### 4.4.2 Radiotherapy to the pelvis damages the surrounding of the pelvis

Live birth rate declined to barely 8% in patients who had received targeted radiotherapy to the pelvis before OTCT, which may be related to poor revascularization activity in ovarian grafts due to (i) a fibrotic reaction in irradiated pelvic tissue (including peritoneum), with poor residual revascularization, and (ii) irradiation of the uterus. The grafting site should be away from the irradiated field, even if it appears healthy at first glance. The radiation dose and zone are key factors that must be considered before OTCT (7).

#### 4.4.3 The challenges of whole ovary cryopreservation and transplantation

Whole ovary cryopreservation and transplantation has been proposed as an alternative to cortical strip freezing, since it could theoretically extend the longevity of ovarian grafts by transplanting the entire follicle pool and avoiding ischemic damage to the ovary thanks to vascular anastomosis (35–38). This requires high levels of surgical expertise, with the risk of losing the entire organ in the event of thrombosis (35, 39–41). Dolmans said they provide unprecedented proof that human ovarian tissue that has undergone whole ovary cryopreservation can resume endocrine and exocrine ovarian function upon autologous OTCT in the form of cortical strips (42).

#### 4.4.4 Clinical translation

Our findings rationalize novel surgical approaches: Orientation control: Positioning grafts with medullary interface against vascularized beds (e.g., ovarian medulla or peritoneal windows) could shorten ischemia by 48-72 hours. This directly aligns with our observed superiority in revascularization (higher CD31^+^/CD34^+^ density) and significantly reduced apoptosis at the medullary interface compared to the cortical surface. Stromal priming: Pretreating cortical surfaces with pro-angiogenic factors may accelerate vascular ingrowth (43).

### 4.5 Limitations and future directions

This investigation acknowledges several methodological constraints that warrant consideration. The primary limitation stems from the restricted patient cohort (n = 5), though the consistency of observed effects across all specimens strengthens the reliability of our observations. Additionally, the murine xenotransplantation model, while providing critical insights into early revascularization dynamics, may not fully recapitulate the complex vascular biology of human pelvic environments. The absence of molecular profiling—particularly of the dynamics within hypoxia-responsive pathways—represents another knowledge gap. The HIF-1α and HIF-2α subunits exhibit distinct temporal regulation in response to acute versus chronic hypoxia, and both regulate key targets like VEGF (44–46). Future mechanistic studies should therefore delineate their respective contributions to the observed revascularization patterns. Furthermore, the study’s focus on early time points (days 3 and 7) leaves the long-term evolution of the observed vascular differences an open question.

To address these limitations and extend our findings, future investigations should prioritize three interconnected directions: First, validation of interface-specific transcriptional profiles through spatial transcriptomics would elucidate molecular drivers of the observed vascular asymmetry. Second, preclinical testing of orientation-optimized transplantation protocols in large-animal models is essential to evaluate translational feasibility. Specifically, directly comparing medullary versus cortical surface oriented downward under controlled conditions would help isolate the intrinsic revascularization capacity of each surface. Third, longitudinal tracking correlating early vascular patterning (days 3-7) with long-term follicular survival metrics will determine whether the observed medullary revascularization advantage translates to improved functional outcomes.

## Data Availability

The original contributions presented in the study are included in the article/supplementary material. Further inquiries can be directed to the corresponding author.
